# Semantic segmentation method of underwater images based on encoder-decoder architecture

**DOI:** 10.1371/journal.pone.0272666

**Published:** 2022-08-25

**Authors:** Jinkang Wang, Xiaohui He, Faming Shao, Guanlin Lu, Ruizhe Hu, Qunyan Jiang

**Affiliations:** Department of Mechanical Engineering, College of Field Engineering and Army Engineering University, PLA, Nanjing, China; Shandong Normal University, CHINA

## Abstract

With the exploration and development of marine resources, deep learning is more and more widely used in underwater image processing. However, the quality of the original underwater images is so low that traditional semantic segmentation methods obtain poor segmentation results, such as blurred target edges, insufficient segmentation accuracy, and poor regional boundary segmentation effects. To solve these problems, this paper proposes a semantic segmentation method for underwater images. Firstly, the image enhancement based on multi-spatial transformation is performed to improve the quality of the original images, which is not common in other advanced semantic segmentation methods. Then, the densely connected hybrid atrous convolution effectively expands the receptive field and slows down the speed of resolution reduction. Next, the cascaded atrous convolutional spatial pyramid pooling module integrates boundary features of different scales to enrich target details. Finally, the context information aggregation decoder fuses the features of the shallow network and the deep network to extract rich contextual information, which greatly reduces information loss. The proposed method was evaluated on RUIE, HabCam UID, and UIEBD. Compared with the state-of-the-art semantic segmentation algorithms, the proposed method has advantages in segmentation integrity, location accuracy, boundary clarity, and detail in subjective perception. On the objective data, the proposed method achieves the highest MIOU of 68.3 and OA of 79.4, and it has a low resource consumption. Besides, the ablation experiment also verifies the effectiveness of our method.

## 1. Introduction

With the rapid social development, production resources are increasingly scarce, and the development and utilization of marine resources become important for human society. Countries around the world are paying more and more attention to the exploitation of ocean resources. More and more researchers begin to pay attention to the field of underwater vision. The current computer vision studies on underwater images mainly include underwater image enhancement [1–3] and underwater object detection [4, 5]. The former is to improve the image quality, and the latter is to perform object recognition and localization. This paper focuses on a new research field of underwater vision, i.e., underwater image semantic segmentation, which aims to classify objects at the pixel level, provide fine target contours for underwater images, and enhance the discrimination between background and objects.

As an important branch of image processing, semantic segmentation [6] divide an image into disjoint and meaningful sub-regions, where the pixels in the same region have a certain correlation and the pixels in different regions have certain differences. That is, semantic segmentation is the process of assigning the same labels to the pixels with the same properties.

**Fig 1:**
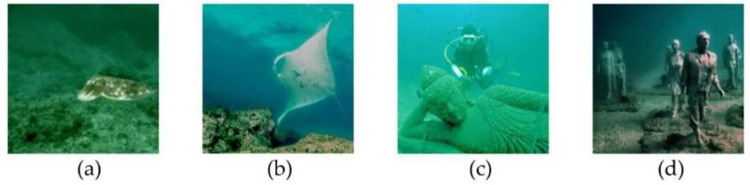
The original underwater images with low image quality.

Due to the complex underwater environment and the scattering and absorption of light by the water medium, underwater images have poor quality. There are problems such as color distortion, low contrast, noise, and uneven illumination, as shown in Fig 1. This poses a great challenge to the integrity and accuracy of underwater image segmentation. In traditional semantic segmentation, some key information is lost because the resolution of the feature graph is reduced by deep convolution networks. The objects of different scales require to combine global and local information, which makes feature extraction difficult. The spatial invariance of CNN leads to a decrease in positioning accuracy and the blurring of the boundary between the object and the background [7].

For low-quality underwater original images, the traditional semantic segmentation methods have poor segmentation results because they cannot deal with complex underwater environment information [8, 9]. To solve the above problems and improve the semantic segmentation effect of underwater images, this paper proposes a new encoder-decoder network structure for the semantic segmentation of underwater images. In this network, the backbone network Resnet obtains the boundary feature map of the images; the densely connected hybrid atrous convolution expands the receptive field; the cascade atrous convolution space pyramid pooling module considers object regions of different scales and optimizes deep semantic features; the context information aggregation mechanism fuses the feature information of the deep network and the shallow network to recover the detailed information of the objects. Experimental results show that compared with the advanced semantic segmentation methods, the proposed method has significant advantages. Also, the effectiveness of each module in the proposed method is verified by the ablation experiment.

The contributions of this paper are summarized as follows:

1. According to the quality degradation characteristics of underwater images, this paper proposes to enhance the images before semantic segmentation, which is original in existing underwater image semantic segmentation methods.
2. The densely connected hybrid atrous convolution can effectively solve the "gridding issue" caused by stacking multi-layer atrous convolution and avoid convolution degradation caused by excessive atrous convolution rate.
3. The cascaded atrous convolution spatial pyramid pooling module can consider the object areas of different sizes, refine the feature maps, and enrich the detailed information of the objects.
4. The context information aggregation mechanism uses a bottom-up method to aggregate the feature information of the deep network and the shallow network to make the object boundary clearer and refine the segmentation results.

This paper is organized as follows. Section 2 briefly reviews the problem of underwater image semantic segmentation and the present research, including the development of semantic segmentation and the latest method of underwater image segmentation. Section 3 introduces the network model proposed in this paper. Section 4 compares our method with other state-of-the-art underwater image segmentation methods and conducts ablation experiments to verify the contribution of each component in the proposed method. Section 5 summarizes the paper and determines the future work.

## 2. Related work

Semantic segmentation [10], a classic computer vision task, provides pixel-level image understanding in a human-perceptual manner by transforming image pixel information into a mask with highlighted regions of interest. Each pixel in the image is assigned a class ID according to the object it belongs to.

Early segmentation algorithms mainly include grayscale segmentation [11], conditional random field [12], etc. These traditional algorithms have drawbacks of high storage overhead and low computational efficiency, and the segmentation effect is not satisfactory. With the continuous development of deep learning, the image semantic segmentation method based on deep learning emerges. Long et al. designed the first real deep learning semantic segmentation model FCN [13], which is improved based on the VGG-16 network [14] adopts a cross-layer method and considers global semantic information and local location information. However, due to a series of pooling operations, the resolution of the feature map is reduced, and the pixel spatial position information is lost, resulting in a rough segmentation effect. In view of the shortcomings of FCN, the Deeplab series of algorithms [15–17] effectively expands the receptive field and captures image context information through an image pyramid [18], atrous convolution [19], and atrous space pyramid pooling [20], obtaining segmentation results with improved spatial accuracy. However, the dense network structure leads to slow segmentation, and the Deeplab series of algorithms do not perform well on small-sized objects. Dilation10 [21] optimized the convolutional structure by appropriately discarding the pooling layer. It effectively slows down the reduction of the resolution of the feature map and increases the receptive field, but the continuity of the local information of the pixel is affected, leading to its sensitivity to unknown deformation. Segnet [22] adopts an encoder-decoder mechanism and restores the spatial position of pixels through up-pooling. It improves the segmentation resolution, but too many network training parameters lead to high computational costs. RefineNet [23] proposed a multi-path optimization network that can effectively obtain the context information of the image and improve the utilization of local and global features. However, the boundary information of the segmentation object will be partially lost. EncNet [24] introduces a context encoding module, which captures the scene contextual semantics and selectively highlights the feature maps related to the categories, making the segmentation results more refined. DenseASPP [25] adopts dense connection based on ASPP and covers a wide range of semantic information. It achieves a good segmentation effect on high-resolution images, but dense connection also leads to the rapid increase of computation overhead. DFANet [26] adopts Xception as the backbone structure and incorporates high-level context into the encoder to achieve an effective balance between segmentation speed and accuracy. DANet [27] adopts a dual-attention network with a self-attention mechanism. It enhances the discriminative ability of scene segmentation feature representation and significantly improves segmentation results. Auto-DeepLab [28] extends NAS [29] to semantic segmentation networks and searches for the optimal network architecture automatically, which gradually improves segmentation accuracy. APCNet [30] uses multiple well-designed adaptive context modules to adaptively construct multi-scale context vectors guided by the global image representation. CANet [31] develops a novel dual-branch dense comparison module to effectively utilize multi-level feature representations from CNNs for dense feature comparison. Also, it adds the attention mechanism to fuse information from different supporting examples. MagNet [32] proposes a multi-scale framework, which gradually refines the segmentation output through multiple amplification levels and spreads information from coarse to fine. The problem of local image blurring is solved, and the segmentation performance is greatly improved. SETR (SEgmentation TRansformer) [33] regards semantic segmentation as a sequence-to-sequence prediction task and encodes images into a series of patches by deploying a pure transformer. The segmentation accuracy is improved by modeling the global context in each layer of the transformer. RobustNet [34] proposes a new case-selective whitening loss, which separates the domain-specific styles encoded in the high-order statistics of feature representation from the domain-invariant content, and selectively deletes only the style information that leads to domain drift. With this loss function, the robustness of the invisible region segmentation network is improved. To sum up, image semantic segmentation methods based on deep learning show great advantages of autonomous learning and classification of robust features, and they achieve better segmentation accuracy and speed than traditional semantic segmentation methods.

Underwater images are difficult to obtain, and compared with conventional images, underwater images are more difficult to segment due to their low resolution, low contrast, uneven illumination, and color distortion. Aiming at the difficulty of underwater image segmentation, Li et al. [35] designed a new weight function and improved the adaptive GACV image segmentation algorithm, which achieves a better segmentation effect on underwater blurred images. Yan et al. [36] solved the problem of underwater image segmentation based on the WOA algorithm. The method can avoid premature convergence and obtain the global optimal solution, which shows a good segmentation effect and high robustness. Chen et al. [37] improved the K-means algorithm to solve the problem of improper determination of the K value in the grayscale quantization process, which reduces the influence of the initial centroid position of the image and greatly improved the segmentation speed and accuracy. Ma et al. [38] proposed the two-dimensional fuzzy Otsu algorithm, which combines the dual advantages of the classic method and fuzzy theory, and the detection effect is more stable and extensive. Zhu et al. [39] combined the local statistical active contour model with co-saliency detection, and the segmentation efficiency and quality are excellent. Liu et al. [40] introduced the unsupervised color correction method into the Deeplab V3+ encoder structure, which improves the segmentation accuracy of the object boundary.

In general, the semantic segmentation of underwater images based on deep learning methods has greatly improved the segmentation quality of underwater images. This paper selects some representative works and briefly introduces their methods, advantages, and disadvantages in Table 1.

**Table 1:** Comparison of semantic image segmentation methods.

Algorithm	Brief methodology	Highlights	Limitations
FCN	Upsampling,Skip layer	The slip layer method considers both global semantic information and local location information.	A series of pooling operations lead to the reduction of feature map resolution and the loss of pixel spatial position information.
Deeplab V3+	Improved atrous convolution, Improved ASPP	Improved atrous convolution and improved ASPP can effectively expand the receptive field, capture context information, and improve the spatial accuracy of segmentation results.	The dense network structure leads to slow segmentation speed, and the segmentation effect is not obvious for small-sized objects.
Dilation10	Atrous convolution, Feature fusion	Properly abandoning the pooling layer can effectively slow down the reduction of feature map resolution while increasing the receptive field.	The continuity of local pixel information is interrupted, and the adaptability to unknown deformation is poor.
Segnet	Encoder-decoder mechanism	Up-pooling is used to restore The spatial position information of pixels and improve the segmentation resolution.	Too many network training parameters lead to high computational costs
RefineNet	Multi-path optimization, Refining module	The multi-path optimization network can effectively obtain the context information of the image, and improve the utilization of local and global features of the image.	The boundary information of the segmentation target will be partially lost.
DFANet	Deep feature aggregation, Lightweight backbones	Incorporate high-level context into the function of coding, and strike an effective balance between segmentation speed and accuracy.	Insufficient ability to obtain image location information.
DANet	Dual attention	The self-attention mechanism is used to integrate the local features of the image and capture the context dependence.	The relationship between each pixel needs to be considered, so the amount of calculation is large.
APCNet	Global-guided local affinity, Adaptive context module	Adaptive construction of multi-scale context representation, combined with local and global representation information to estimate robust weights, greatly improves the semantic segmentation effect.	The network structure is complex, the number of parameters is large, and the calculation cost is high.
CANet	Dense Comparison Module, Iterative Optimization Module	The multi-level feature representation from CNN is effectively used for intensive feature comparison, and an attention mechanism is added to fuse information from different support examples.	Insufficient ability to obtain image location information.

However, the simple semantic segmentation of low-quality underwater images cannot achieve good results. Based on the above literature, this paper enhances the quality of underwater images first and then proposes the semantic segmentation method. First, this paper adopts densely connected hybrid atrous convolution, which can cover a larger receptive field and more effectively maintain the integrity of feature information and reduce information loss than simple multi-layer hole convolution such as Deeplab series algorithms. Secondly, the cascaded atrous convolutional spatial pyramid pooling module refines the feature map and shares the multi-scale target information by fusing the image features under different receptive fields. This is novel for the existing studies. Finally, the context information aggregation network is put forward. By fusing the feature information in the deep network and the shallow network, the details are richer and the edge details are more fully captured.

## 3. The recommended method’s overview

**Fig 2:**
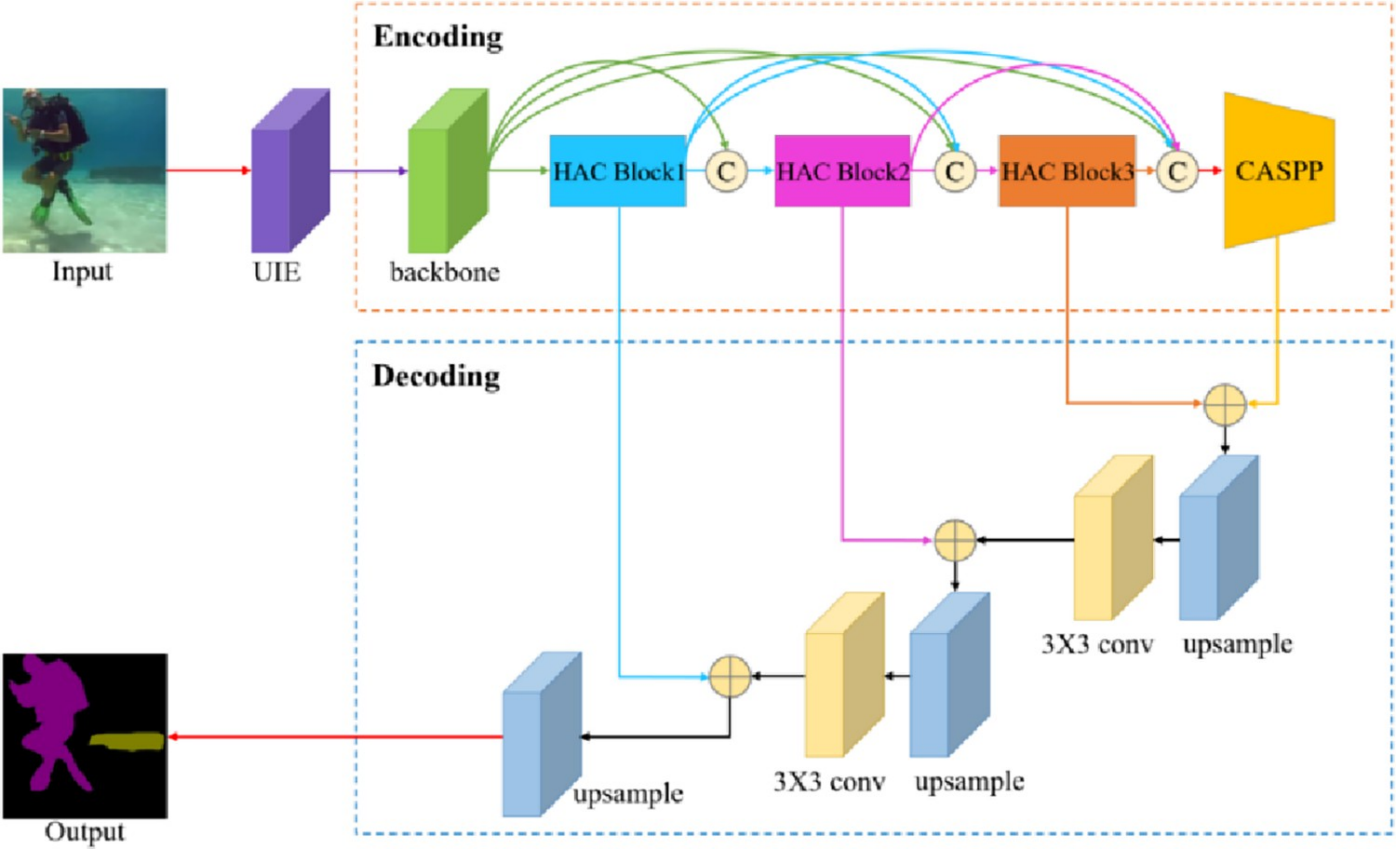
The pipeline of the proposed method.

To solve the difficulties in the semantic segmentation of underwater images, this paper proposes an encoder-decoder architecture for underwater image semantic segmentation based on context information fusion. The flowchart of the algorithm is shown in Fig 2. Firstly, a multi-space transformation underwater image enhancement (UIE) module based on weighted fusion is proposed to enhance the original underwater images. Then, the enhanced images obtain the boundary feature map through the backbone network Resnet [41] with 50 network layers, 1 × 1 and 3 × 3 filters, and a stride of 2. The feature maps are sent to the densely connected hybrid atrous convolution module (DCHAC) to expand the neuron receptive field and slow down the resolution reduction [42]. The HAC block contains three 3 × 3 atrous convolutions, and the atrous rates of the atrous convolutions are 1, 2, and 5, respectively. Next, the cascaded atrous convolutional spatial pyramid pooling module (CASPP) integrates boundary features of different scales to enrich object details, which contains two 1 × 1 convolutions and three 3 × 3 atrous convolutions with atrous rates of 5, 9, and 13 respectively, and the number of filters is 256. Finally, the context information aggregation decoder (CIAD) is proposed to fuse the features of the shallow network and the deep network, which extracts rich contextual information, and uses 3 × 3 convolution to fine-tune the features, which fully grasps the edge details and greatly reduces information loss. Finally, the segmentation effect map is obtained by bilinear interpolation with two times up-sampling. The size of the segmentation map is consistent with that of the original image.

### 3.1 Multi-space transformation underwater image enhancement module

**Fig 3:**
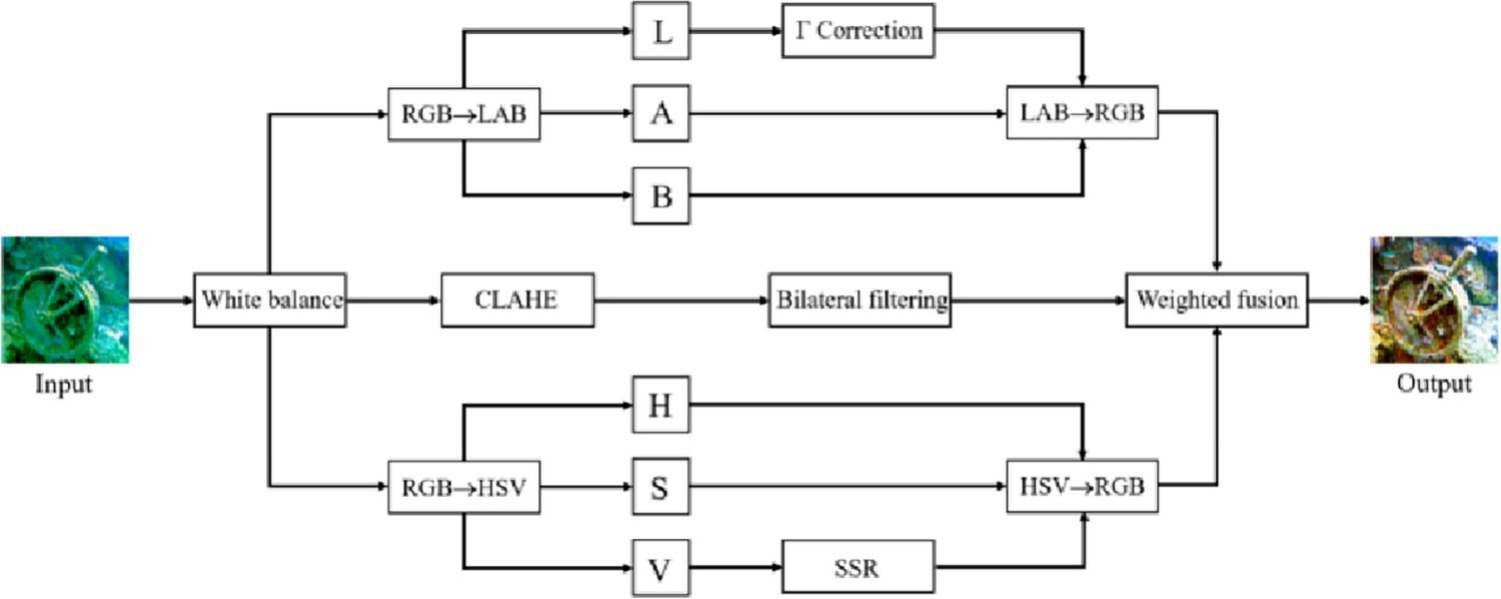
The flowchart of the proposed underwater image enhancement algorithm.

For underwater images with low resolution, low contrast, uneven illumination, and color distortion, this paper first enhances the quality of the original underwater images. The flowchart of the multi-space transformation underwater image enhancement algorithm is shown in Fig 3.

First, white balance processing is performed to correct the color cast of the image [43]. The purpose of white balance is to eliminate or reduce the color cast caused by the refraction of light. In this process, the gray world method is exploited to remove the blue-green appearance and compensate the red channel of the image to eliminate the red artifact. The red channel compensation formula is as follows:

$$I_{rc}(A)=I_{r}(A)+({\bar{I}}_{g}-{\bar{I}}_{r})\times (1-I_{r}(A))\times I_{g}(A)$$ (1)

where, *I*_*rc*_(*A*) is the red channel value at point A after compensation; *I*_*r*_(*A*) and *I*_*g*_(*A*) are respectively the values of the red and green channels at point A of the original image; ${\bar{I}}_{g}$ and ${\bar{I}}_{r}$ are respectively the average values of the green and red channels of the original image.

Then, the following operations are performed on the images after white balance processing:

1. Convert the image from RGB space to LAB space and process the L channel with γ correction and then convert the image back to RGB space. γ correction can increase the scale of the region of interest in the image. It has a good dynamic compression effect and can adjust the overall brightness of the image by using two variable parameters *γ* and *c*. The expression of γ transform is as follows,

$$I_{1}=cI_{i}^{\gamma }$$ (2)

where, *I*_*i*_ represents the intensity of the input image; *I*_1_ represents the intensity of the output image; *γ* and *c* are used to adjust the shape of the γ function.

2. The contrast-limited adaptive histogram equalization algorithm (CLAHE) [44] and bilateral filtering [45] are carried out in the RGB space. White-balanced images still have noise and low contrast. CLAHE can obtain enhanced images with uniform brightness, and bilateral filtering can remove noise and preserve edges to improve image clarity. The output after this operation is denoted as *I*_2_.
3. Convert the image from RGB space to HSV space and process the V-channel with single-scale retinex (SSR) [46] and then convert the image back to RGB space. The HSV color space is more in line with the visual characteristics of human beings. Converting the underwater image to HSV space, keeping the hue channel H component and saturation channel S component of the image unchanged, and enhancing the luminance value channel V with SSR combined with guided filtering [47] can help to obtain images suitable for human observation. The output after this operation is denoted as *I*_3_.

**Fig 4:**
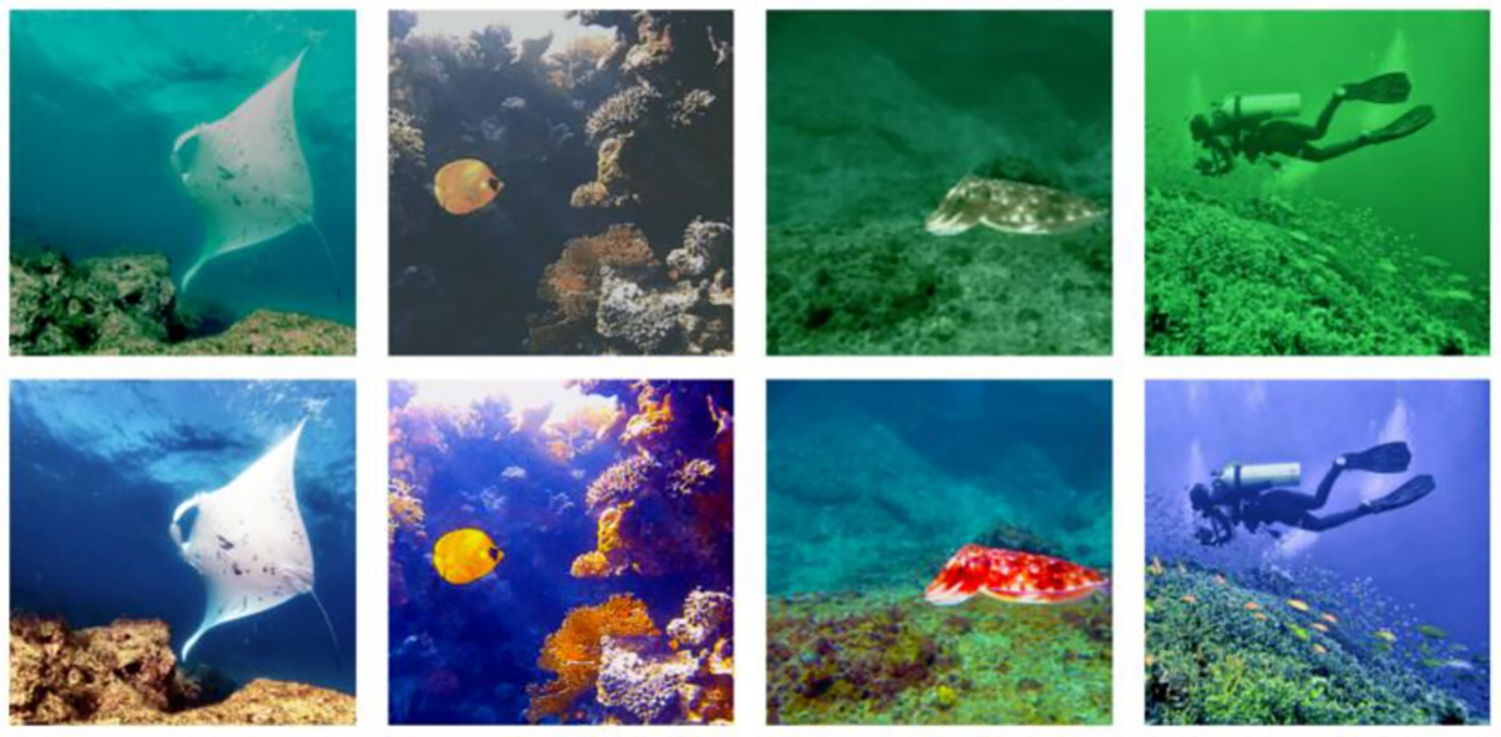
Comparison of underwater image enhancement effect. The upper line shows the original underwater images, and the lower line shows the enhanced images.

The outputs *I*_1_, *I*_2_, and *I*_3_ are weighted and fused to obtain the following results:

$$I_{0}=aI_{1}+bI_{2}+cI_{3}$$ (3)

where a, *b*, and *c* are control parameters, and their sum is 1. Through experiments, it is indicated that when a = 0.4, *b* = 0.3, and *c* = 0.3, the image enhancement effect is the best. Fig 4 shows the effect comparison before and after enhancement.

### 3.2 Densely connected hybrid atrous convolution module

**Fig 5:**
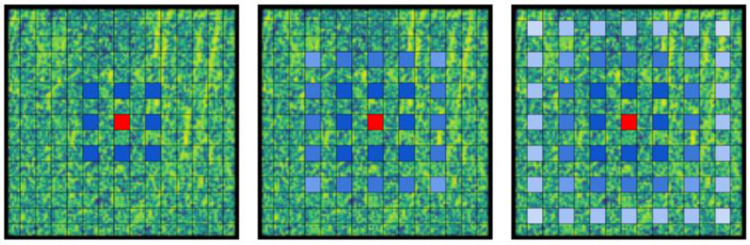
Covering effect of three-layer atrous convolution with equal atrous rate.

Deeplab series networks use multi-layer continuous atrous convolution for feature extraction. It enlarges the receptive field but causes the "gridding issue" [48]. This is because after using multi-layer atrous convolution, the input samples become sparse, which leads to the loss of some local information and the neglect of the overall continuity information of the feature map. As shown in Fig 5, the three-layer atrous convolution with a size of 3×3 and a rate of 2 is taken as an example. The continuous atrous convolution covers the incomplete area of the feature map, and the extracted features are lost or even wrong. Thus, it is difficult to achieve a good segmentation effect by using sparse pixel information.

**Fig 6:**
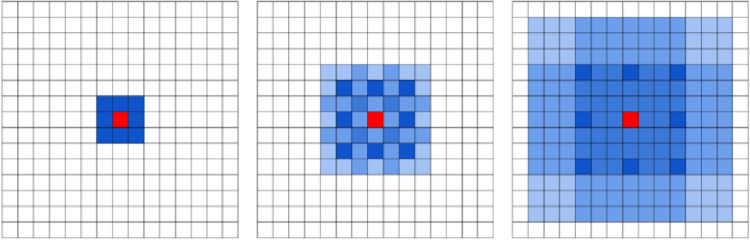
Covering effect of hybrid atrous convolution with unequal atrous rate.

To solve this problem, this paper adopts hybrid atrous convolution with different atrous rates. A small atrous rate is used to extract local information, and a large atrous rate is used to extract long-distance information. This can help to obtain wider regional information and improve the information utilization rate while expanding the receptive field. As shown in Fig 6, the hybrid atrous convolution with atrous rates of 1, 2, and 3 is taken as an example. The coherent receptive field completely covers the whole feature map, which ensures the integrity of information extraction. The hybrid atrous convolution structure is expressed as follows.

$$M_{i}=\max[M_{i+1}-2r_{i},M_{i+1}-2(M_{i+1}-r_{i}),r_{i}]$$ (4)

where, *r*_*i*_ is the atrous rate of layer *i*, and *M*_*i*_ is the maximum atrous rate of layer *i*. For the convolution kernel size of k*k, *M*_*r*_≤*k* should hold so that all the receptive fields can be covered.

This paper connects three hybrid atrous convolution blocks through dense connection to encode multi-scale semantic information. Based on this, the final output features cover a large range of semantic information and cover information coding in a very dense way.

### 3.3 Cascaded atrous convolutional spatial pyramid pooling module

**Fig 7:**
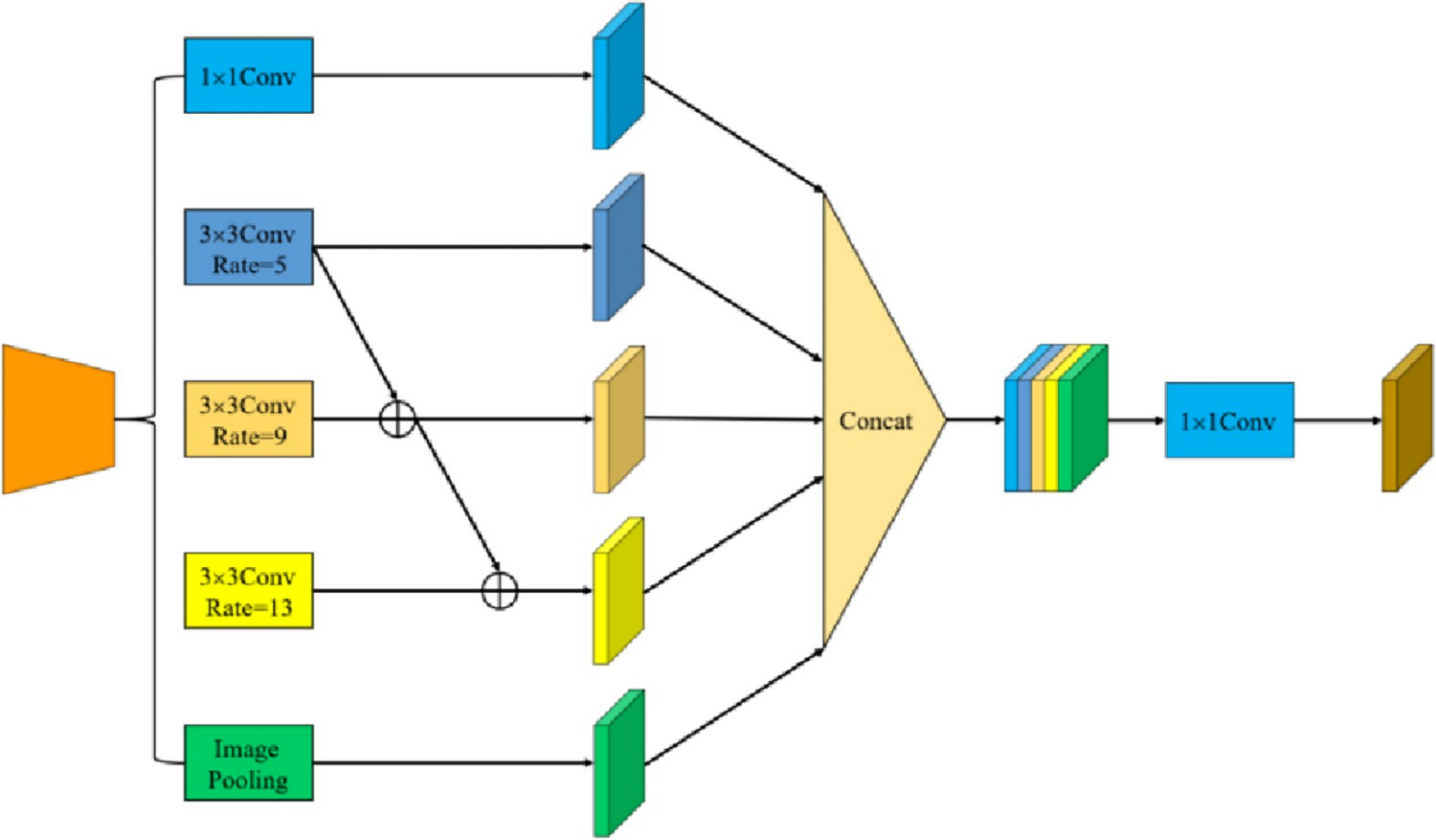
The structure of the CASPP module.

For low-quality underwater images, layer-by-layer convolution gradually reduces the resolution of the feature map, which will lead to the loss of segmentation details and the weakening of the correlation of each pixel. To address this issue, this paper parallels three atrous convolution blocks with atrous rates of 5, 9, and 13 to refine the feature map. The output feature maps under different receptive fields are cascaded and fused to share multi-scale target information, which further improves the relevance and continuity of global pixel information. Finally, the obtained five feature maps are fused in the channel dimension, which involves the object areas of different sizes, enriches the object details, and helps to obtain higher-level image semantic information. The structure of the CASPP module is shown in Fig 7.

### 3.4 Context information aggregation decoder

The feature maps of different network depths have different characteristics. The shallow feature map is large in scale and has more feature information, which can be used to easily distinguish the object. However, due to the limitation of convolution kernels and computing resources, the shallow network can only extract local features with detailed information, such as color and texture within a small receptive field. After layer-by-layer convolution and down-sampling, the resolution and the size of the deep network feature map are reduced, and the receptive field is enlarged. The deep network feature can extract relatively clear high-level semantic information such as location and category in a global scope. However, due to the lack of geometric space detail, the accuracy of edge segmentation is not enough. The fusion of feature information in the deep network and the shallow network can greatly reduce the loss of image feature information, leading to richer detail information richer and clearer edge segmentation results [49, 50].

**Fig 8:**
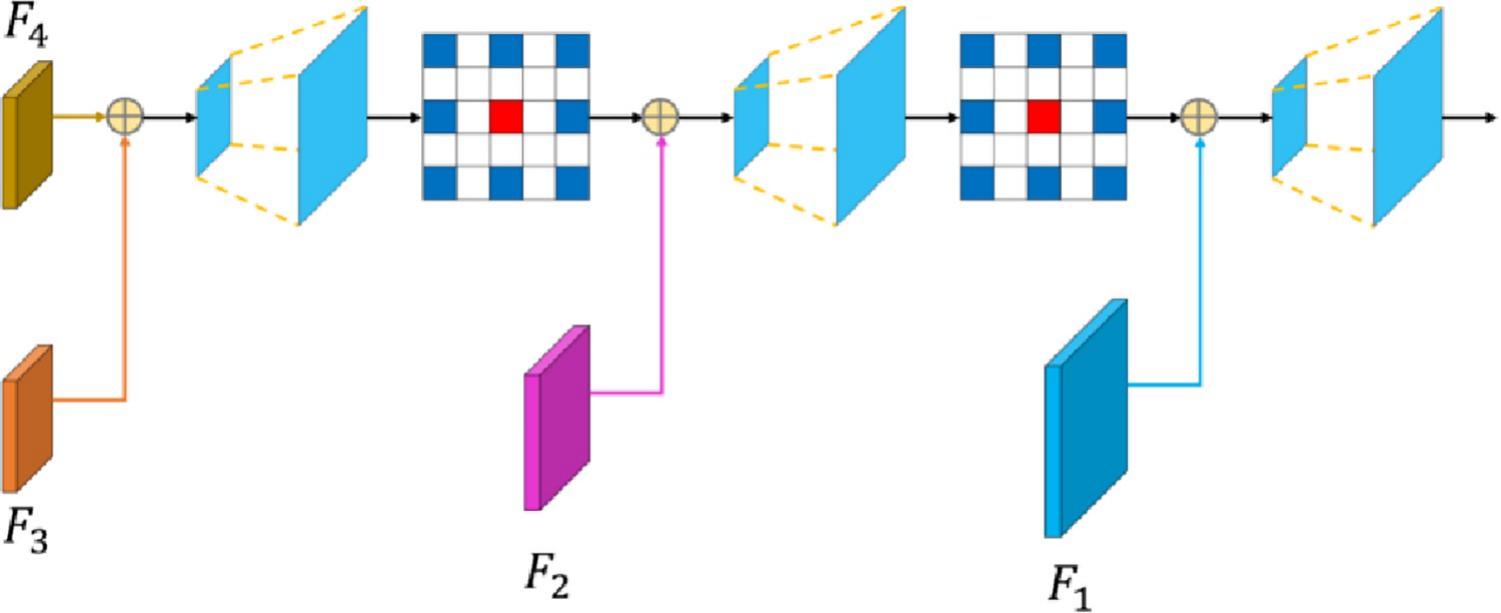
The structure of the context information aggregation decoder.

The structure of the context information aggregation decoder is shown in Fig 8. First, the feature map F4 output by the CASPP module and the feature map F3 extracted by the hybrid atrous convolution block 3 are added pixel by pixel. Then, the aggregated feature map is upsampled two times to increase the resolution of the feature map, and the atrous convolution with an atrous rate of 2 is used to combine the feature information of adjacent pixels to refine the upsampling features. Next, the above operations are repeated with the feature map F2 extracted by the hybrid atrous convolution block 2. The obtained feature map is added with F1 extracted by the hybrid atrous convolution block 1, and the result is finally upsampled four times to aggregate the whole network context. The context information aggregation decoder gradually merges the deep semantic information with the shallow edge line, shape position, and other detailed information. This helps to capture clear object boundary information, refine segmentation results, and effectively improve the accuracy of object segmentation.

## 4. Experimental results

### 4.1 Dataset

**Fig 9:**
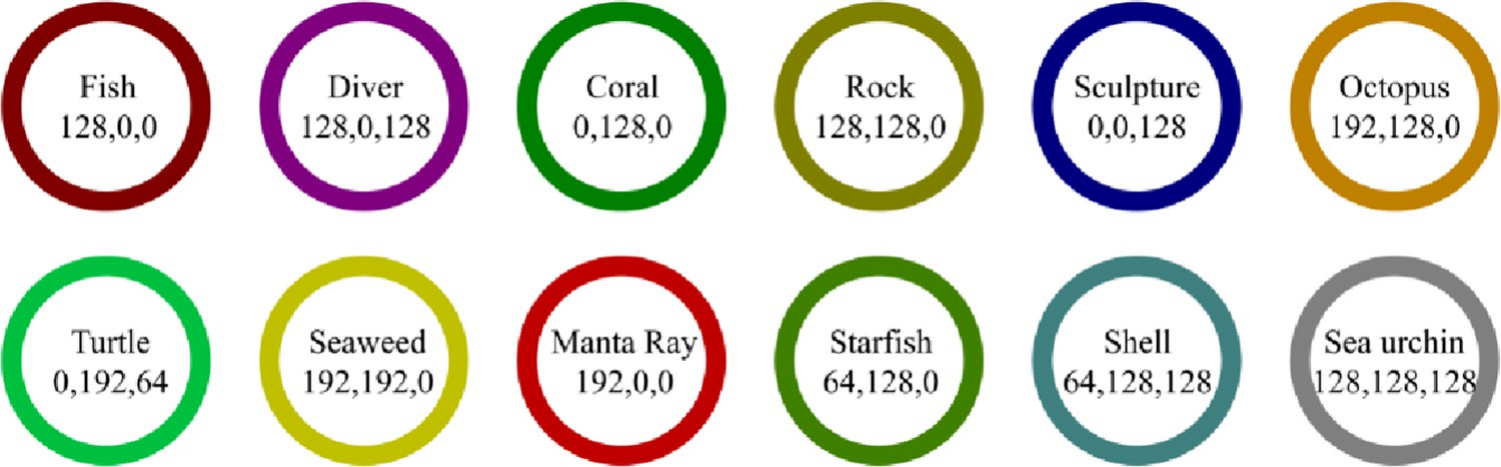
The color representation of labeled categories.

To complete the research on semantic segmentation of underwater images, this paper combined several open-source underwater image datasets to construct an underwater image segmentation dataset, and the composition of the dataset is shown in Table 2. This paper selected 3,000 images for annotation. The “Resize” operation was used to cluster large-resolution images, and pixel interpolation was performed on small-resolution images. Then, the image information was extracted, and the pixels were rearranged to the resolution of 512×512 to achieve a uniform distribution. 3,000 of the images were marked by manual labeling with the LabelMe software. The labeling includes 12 categories: fish, diver, coral, rock, sculpture, octopus, turtle, seaweed, manta ray, starfish, shell, and sea urchin. The labeling format of the COCO dataset [51] was used. To facilitate the research of semantic segmentation, the mask images of semantic segmentation similar to those in the PASCAL VOC 2012 dataset were provided [52]. The ratio of the training set to the testing set is 4:1. The category statistics are shown in Fig 9.

**Table 2:** Summary of underwater datasets.

Dataset	Quantity	Marked quantity	Resolution ratio	Scene distribution	Source
RUIE [53]	4000	500	400×300	Scallop, kelp, sea urchin	Dalian University of Science and Technology
HabCam UID [54]	10465	2300	2720×1024	Fish, scallop	CVPR AAMVEM studio
UIEBD [1]	950	200	Multiresolution	Diver, statue, marine life	City University of Hong Kong

### 4.2 Experimental setting

Our proposed method was tested in PyTorch software on a computer equipped with Intel Core i7-6700U CPU (4.00 GHz), NVIDIA GeForce RTX 4000, 8 GB DDR3 memory, and running Windows 10 64-bit operating system.

As for training parameter settings, the initial learning rate was set to 0.01, and the model was trained for 30 epochs using the learning rate cosine decay strategy. The network was optimized using SGD with momentum. The momentum parameter was set to 0.9, the weight decay regularizer was set to 0.00005, and the batch size was set to 16.

### 4.3 Performance evaluation index

This paper used *F*1, *OA*, *IOU*, and *MIOU* as comprehensive evaluation indexes to evaluate the segmentation effect of our proposed method. These indexes are widely used in many studies [55, 56]. The ground truth was obtained through manual labeling. *TP* indicates the number of samples whose prediction results are true and the ground truths are true; *FN* indicates the number of samples whose prediction results are false and the ground truths are true; *FP* indicates the number of samples whose prediction results are true and the ground truths are false; *TN* indicates the number of samples whose prediction results are false and the ground truths are false. *R* represents the recall rate, and its calculation is shown in Formula (5). *P* represents the precision, and its calculation is shown in Formula (6). *F*1 comprehensively evaluates the recall rate and the precision, and its calculation is shown in Formula (7).


$$R=\frac{TP}{TP+FN}$$ (5)

$$P=\frac{TP}{TP+FP}$$ (6)

$$F1=\frac{2R\times P}{R+P}$$ (7)

*OA* represents overall accuracy, and it indicates the proportion of correctly classified samples to all samples. The calculation formula is shown as follows.


$$OA=\frac{TP+TN}{TP+FN+FP+TN}$$ (8)

*IOU* represents the intersection over the union between the predicted value and the real value of each category, and its calculation is shown in Formula (9). *MIOU* is the average value of IOU in all different categories, and its calculation is shown in Formula (10).

$$IOU=\frac{TP}{FN+FP+TP}$$ (9)

$$MIOU=\frac{1}{k+1}\sum_{i=0}^{k}\frac{TP}{FN+FP+TP}$$ (10)

where *k* represents the number of categories, and there are *k*+1 categories if the background is included.

### 4.4 Analysis of experimental results

#### 4.4.1 Subjective evaluation

**Fig 10:**
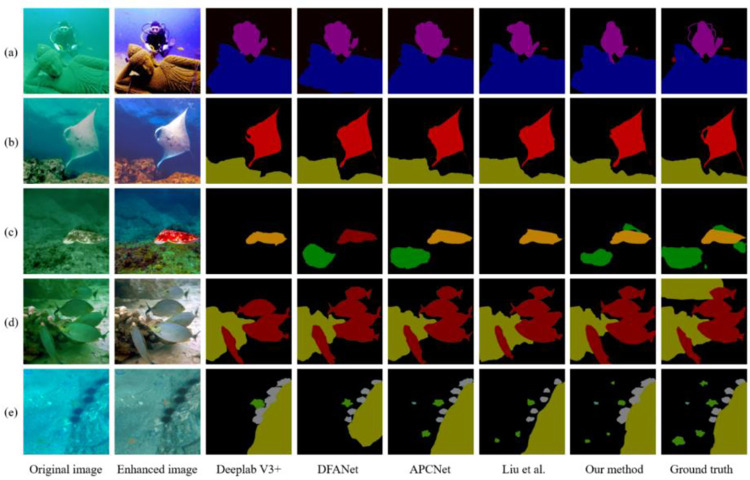
Qualitative comparisons on colored cast underwater images. From left to right are original images, the enhanced images, and the results generated by Deeplab V3+, DFANet, APCNet, the method proposed by Liu et al., our method, and the ground truth.

**Fig 11:**
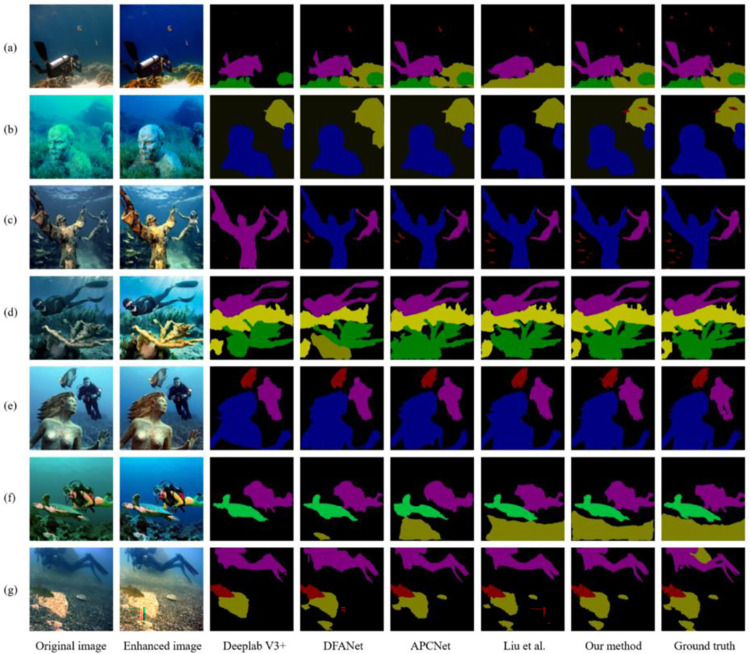
Qualitative comparisons on clear underwater images. From left to right are the original images, the enhanced images, and the results generated by Deeplab V3+, DFANet, APCNet, the method proposed by Liu et al., our method, and the ground truth.

To intuitively show the effectiveness of our proposed method, the comparison results between our method and four state-of-the-art underwater image semantic segmentation methods (Deeplab V3+, DFANet, APCNet, and Liu et al. [40]) on the composite dataset are shown in Figs 10 and 11. From left to right are the original image, the enhanced image, and the results generated Deeplab V3+, DFANet, APCNet, Liu et al. [40], our method, and the ground truth.

Due to the color cast, the contrast of the underwater image is reduced. From the experimental results in Fig 10, it can be seen that our method achieves the best segmentation effect on underwater images with a serious color cast. Vertically, Deeplab V3+ misses some image information due to the decrease in contrast, and it cannot fully segment each category. The rock in image (c), the fish on the upper side in image (d), the starfish, and the shell in image (e) are not segmented correctly, and the edges are inaccurately positioned. DFANet performs slightly better than Deeplab V3+, but incorrectly classifies octopuses in image (c) and fails to capture the smaller starfish and shells in image (e). APCNet’s segmentation range is too large for already captured object categories, such as the expansion of the diver’s segmentation boundary in image (a). Compared with the previous methods, the algorithm proposed by Liu et al. [40] achieves a better segmentation effect, but it cannot achieve a good segmentation effect for images with low contrast such as image (c). Compared with other algorithms, our method has the advantages of complete object segmentation and accurate positioning. Although there are problems of misjudgment and unclear boundaries, the segmentation results obtained by our method are the closest to the ground truth. Horizontally, for images with blurred boundaries such as images (a), (b), and (d), the segmentation result of the proposed method is clear and closest to the ground truth; For images with low contrast such as image (c) and (e), the effect of image enhancement is obvious. Our method can accurately locate some small targets that are easy to miss, while other methods have different degrees of misjudgments and omissions.

Here, a subjective visual comparison was performed on the segmentation effect of the underwater images with higher resolution. Vertically, the segmentation effect of Deeplab V3+ is not ideal. There are different degrees of missed classification, and the classification result is not accurate. For example, the sculpture in image (c) is misclassified as a diver. DFANet achieves a slightly better segmentation effect, but there are also false classifications, e.g., the small sculpture in image (b) and the coral in image (d) are both misclassified as a rock. The segmentation effect of APCNet and Liu et al. [40] is better, but the details still need to be improved. For example, the two small fishes in front of the rock in image (b) cannot be segmented, and the segmentation shapes of the starfish and sea urchins in image (g) are quite different from the ground truth. Compared with the above four methods, our method performs better with clear boundaries and accurate positioning, but there are still some problems, such as inconspicuous details and incomplete object segmentation. Horizontally, although the segmentation effect of our method is the closest to the ground truth, the difference with other methods is not obvious, which is also consistent with our expectations. It can be seen that image enhancement plays an important role in the semantic segmentation of underwater images.

Overall, our method is the best in terms of segmentation completeness, localization accuracy, boundary definition, and detail.

#### 4.4.2 Objective evaluation

The *F*1, *OA*, *IOU*, and *MIOU* evaluation indexes mentioned in Section 4.2 were adopted to evaluate the segmentation accuracy. The larger the value of the four indexes, the better the segmentation effect. The *IOU* and *F*1 test results for each category are shown in Table 3, and the *MIOU* and *OA* test results for all categories are shown in Table 4. The best results are marked in bold. It can be seen that our method achieves the largest *IOU* and *F*1 values in most categories and obtains the highest *MIOU* and *OA* in all categories. The segmentation effect of our method is the best in terms of the objective evaluation, which is consistent with the subjective evaluation. It should be noted that although our method has no significant improvement in objective results, it has a great improvement in subjective visual perceptions compared with other algorithms.

**Table 3:** Comparison of *IOU* and *F*1 values between our method and the other four state-of-the-art methods on the underwater dataset. The best results are marked in bold.

Target category	Deeplab V3+		DFANet		APCNet		Liu et al.		Our method	
	IOU	F1	IOU	F1	IOU	F1	IOU	F1	IOU	F1
Fish	63.4	75.2	60.2	77.2	66.1	76.3	64.4	76.9	**70.5**	**78.0**
Diver	63.2	73.7	61.4	75.8	63.0	75.2	63.1	76.2	**65.4**	**77.5**
Coral	61.1	74.1	**66.2**	75.4	63.5	76.4	63.8	**76.5**	65.6	76.3
Rock	59.8	80.2	59.1	80.5	61.4	80.2	61.2	80.1	**63.4**	**81.2**
Sculpture	62.4	74.5	62.1	76.1	64.2	**78.4**	63.0	77.8	**69.5**	77.4
Octopus	64.6	72.3	61.8	75.1	63.6	75.2	66.3	74.9	**67.1**	**75.7**
Turtle	**67.0**	75.9	60.3	77.7	63.7	77.1	65.8	77.8	66.7	**78.6**
Seaweed	60.3	79.1	65.7	79.3	66.3	79.6	67.1	**79.9**	**68.9**	79.4
Manta Ray	62.8	78.7	62.9	79.4	67.5	79.8	**68.1**	80.1	67.8	**81.1**
Starfish	61.1	79.4	61.7	80.4	67.0	80.5	68.4	80.4	**70.1**	**81.5**
Shell	60.2	75.1	60.9	75.7	64.1	76.3	62.6	76.5	**65.9**	**77.3**
Sea urchin	60.7	79.5	64.5	80.6	64.9	80.7	63.4	80.7	**67.8**	**82.0**

**Table 4:** Comparison of *MIOU* and *OA* values between our method and the other four state-of-the-art methods on the underwater dataset. The best results are in bold.

Method	Deeplab V3+	DFANet	APCNet	Liu et al.	Our method
MIOU	61.3	63.2	65.5	64.9	**68.3**
OA	77.5	78.0	78.6	78.5	**79.4**

Table 5 shows the comparison results of parameters, FLOPs, and FPS between our method and other algorithms on the underwater dataset. It can be seen that our method has relatively few parameters, small FLOPs, and less occupation of computing resources. Meanwhile, the operating speed is relatively fast, and the FPS reaches 125. Generally, our method has a good balance between segmentation accuracy and speed.

**Table 5:** Comparison of execution speed of different algorithms on the CUOID dataset.

Method	Input size	Parameters(M)	FLOPs(G)	FPS
Deeplab V3+	513×513	213.1	~	0.4
DFANet	512×1024	7.8	1.7	160
APCNet	512×1024	~	~	~
CANet	640×360	19.7	6.2	72
Our method	512×512	9.6	1.8	125

### 4.5 Ablation experiment

The contribution of each module in our method to algorithm performance was validated on the underwater image dataset. The ablation experiment results of adding modules DCHAC, ACSPP, CIA decoder, and UIE to the feature extraction network framework are presented in Table 6, where “Decoder” represents the decoder structure of DeeplabV3+. The experimental results show that the addition of each module has a certain effect, and the accuracy of image semantic segmentation has been improved. Among them, the addition of the UIE module has the greatest impact on the enhancement of underwater image semantic segmentation. And the objective evaluation index reaches the highest when the four modules are added, indicating that the four modules are indispensable, and the combined effect is the best for underwater image segmentation.

**Table 6:** The contribution of adding DCHAC, ACSPP, CIA decoder, and UIE to the objective evaluation performance. The best results are marked in bold.

Baseline	DCHAC	CASPP	Decoder	CIA Decoder	UIE	MIOU
✓	✓		✓			60.2
✓	✓			✓		61.9
✓	✓	✓	✓			64.3
✓	✓	✓		✓		65.7
✓	✓	✓		✓	✓	**68.3**

### 4.6 Failure examples

**Fig 12:**
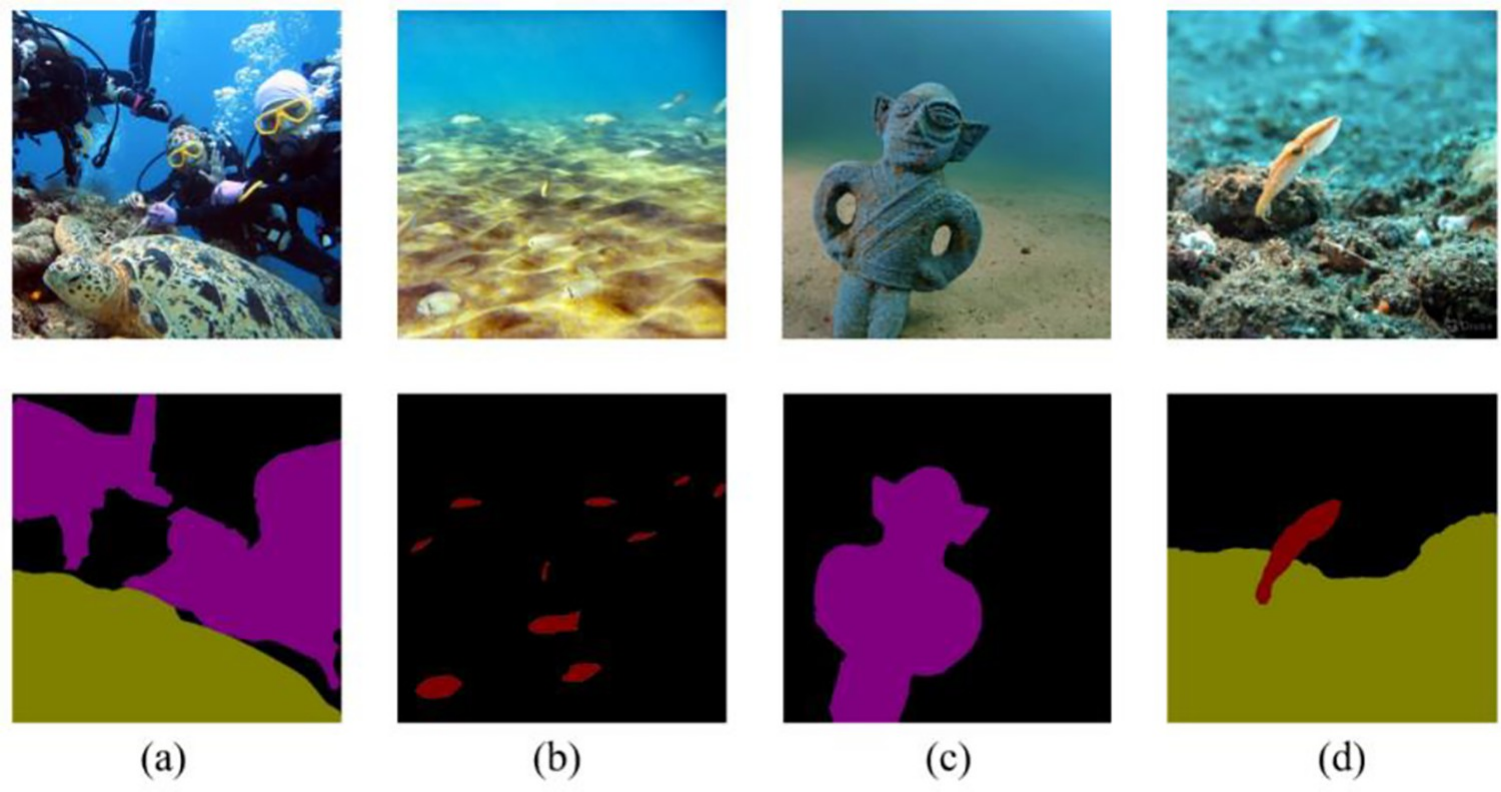
Failure examples of the proposed method.

Fig 12 shows the failure examples of our proposed network. Since our segmentation network segments different objects by pixel region, some objects of different classes but similar shapes are difficult to distinguish. In addition, the change of scale leads to poor segmentation results for some small objects. As shown in Fig 12, the turtle of (a)is mistakenly detected as a rock due to the insignificant pixel difference, in (b) many little fish are not detected due to the light and shadow phenomenon caused by the refraction of the seawater, the humanoid sculpture in (c) was mistakenly detected as a diver, the octopuse in (d) is mistakenly identified as marine fish due to indistinguishable shapes.

## 5. Conclusion and future work

According to the characteristics of underwater images, this paper proposes an underwater image semantic segmentation method based on encoder-decoder architecture. Firstly, the image enhancement based on multi-spatial transformation is performed to enhance the original images. Then, the densely connected hybrid atrous convolution effectively expands the receptive field and slows down the speed of resolution reduction. Subsequently, the cascaded atrous convolutional spatial pyramid pooling module integrates boundary features of different scales to enrich target details. Finally, the context information aggregation decoder fuses the features of the shallow network and the deep network to extract rich contextual information, which greatly reduces information loss.

To verify the effectiveness of the proposed method, this paper established a comprehensive underwater image dataset, and our method was compared with four mainstream semantic segmentation methods through subjective and objective evaluations. The experimental results show that our method has obvious advantages in both subjective perception and objective data, and it outperforms the existing methods in terms of segmentation integrity, positioning accuracy, boundary clarity, and details. Meanwhile, ablation experiments verify the contribution of each component in our method to the final segmentation performance.

The method proposed in this paper improves the segmentation effect, but there are still misjudgments and omissions for objects with little discrimination. Our future work will focus on this problem to reduce the occurrence of misjudgments and omissions. Also, we will combine object detection and image enhancement to systematically analyze and improve underwater image processing.
